# Numerical Studies on the Failure Process of Heterogeneous Brittle Rocks or Rock-Like Materials under Uniaxial Compression

**DOI:** 10.3390/ma10040378

**Published:** 2017-04-01

**Authors:** Songfeng Guo, Shengwen Qi, Yu Zou, Bowen Zheng

**Affiliations:** 1 Key Laboratory of Shale Gas and Geoengineering, Institute of Geology and Geophysics, Chinese Academy of Sciences, Beijing 100029, China; guosongfeng@mail.iggcas.ac.cn (S.G.); zouyu@mail.iggcas.ac.cn (Y.Z.); zhengbowen@mail.iggcas.ac.cn (B.Z.); 2 University of Chinese Academy of Sciences, Beijing 100049, China

**Keywords:** material heterogeneity, progressive failure, stress distribution, uniaxial compression

## Abstract

In rocks or rock-like materials, the constituents, e.g. quartz, calcite and biotite, as well as the microdefects have considerably different mechanical properties that make such materials heterogeneous at different degrees. The failure of materials subjected to external loads is a cracking process accompanied with stress redistribution due to material heterogeneity. However, the latter cannot be observed from the experiments in laboratory directly. In this study, the cracking and stress features during uniaxial compression process are numerically studied based on a presented approach. A plastic strain dependent strength model is implemented into the continuous numerical tool—Fast Lagrangian Analysis of Continua in three Dimensions (FLAC^3D^), and the Gaussian statistical function is adopted to depict the heterogeneity of mechanical parameters including elastic modulus, friction angle, cohesion and tensile strength. The mean parameter μ and the coefficient of variance (*h*_cv_, the ratio of mean parameter to standard deviation) in the function are used to define the mean value and heterogeneity degree of the parameters, respectively. The results show that this numerical approach can perfectly capture the general features of brittle materials including fracturing process, AE events as well as stress-strain curves. Furthermore, the local stress disturbance is analyzed and the crack initiation stress threshold is identified based on the AE events process and stress-strain curves. It is shown that the stress concentration always appears in the undamaged elements near the boundary of damaged sites. The peak stress and crack initiation stress are both heterogeneity dependent, i.e., a linear relation exists between the two stress thresholds and *h*_cv_. The range of *h*_cv_ is suggested as 0.12 to 0.21 for most rocks. The stress concentration degree is represented by a stress concentration factor and found also heterogeneity dominant. Finally, it is found that there exists a consistent tendency between the local stress difference and the AE events process.

## 1. Introduction

The failures of brittle rocks or rock-like materials under compression have been proved to be a process of crack initiation, propagation and coalescence [1,2,3,4,5,6,7]. It is widely accepted that such characteristics are mainly resulted from the material heterogeneity due to different types of strong or weak minerals and microdefects in meso-level. In response to external forces or displacements, the induced local stress is not homogeneous because of non-uniform deformation of the heterogeneous microelements in the materials. When the concentrated stresses in some local elements (e.g., weak minerals or grain boundaries) reach the strength threshold, these weak minerals or grain boundaries would be damaged either in shear or tension and crack initiation occurs. The deformation modulus, cohesive and tensile strength drop to residual level for the damaged weak elements, and the stress and deformation through the materials are redistributed instantaneously. As the external forces or displacements increase, further damages would occur when the redistributed local stress reaches the strength threshold in other positions and these processes will repeat until the overall ruptures occur following crack coalescences.

It is shown that the stress redistribution induced by material heterogeneity plays a key role in the failure process. Researchers have conducted a number of experiments in lab or acoustic emission monitoring in field to identify the stress level during fracturing process. It has been indicated that the crack initiation starts at about 0.3–0.6 times of peak stress for most brittle rocks under compression [2,3,4,5,6,7]. Nicksiar and Martin (2013) [8] concluded more precisely that the crack initiation stress to peak stress ratio ranged from 0.42 to 0.47 regardless of the material properties in uniaxial compression after analyzing the stress–strain curves from 376 laboratory tests carried out on samples of igneous, sedimentary and metamorphic rocks. Nevertheless, some other reports showed that the ratio can be out of the range, e.g., as small as 0.13 for the granite at the ZEDEX tunnel [9]. Moreover, the material heterogeneity also causes scatter of peak stress [10,11,12]. The Gaussian distribution function was reached to represent the uniaxial compressive strength scatter of both sound and defective Matinenda sandstone [10].

Except for these valuable but a little rough achievements, it experiences of great difficulties to study the stress disturbance and redistribution during the failure process from the lab experiments and field monitoring. One can hardly obtain detailed insights from experimental studies, e.g., the stress disturbance and transverse, changes of independent component properties due to damage and its influence on adjacent components. Numerical simulation approaches have great advantages through which the stress, displacement and other variables can be recorded and analyzed in detail. 

In this study, the crack features and stress characteristics in failure process of brittle materials with various heterogeneity degrees under uniaxial compression are analyzed quantitatively and qualitatively in detail based on numerical approaches. The framework is: the existing numerical tools to study material heterogeneity are briefly reviewed and the approach to be used is introduced in Section 2, the description of the heterogeneous material models and numerical tests are presented in Section 3, the process based on the numerical tests is depicted in detail and compared with general observation in lab tests, and the influence of heterogeneity on the fracturing and stress concentration process is analyzed in Section 4, and discussion and conclusion are presented in Section 5 and Section 6, respectively.

## 2. The Numerical Approach

### 2.1. Brief Review of the Existing Numerical Approaches for Brittle Fracturing of Materials

The numerical methods are typically classified as continuum methods and discrete methods, and also hybrid continuum/discrete methods [13]. 

The discrete element method (DEM) represents modeling techniques that treat the material directly as an assembly of separate blocks or particles, and allow finite displacements and rotations of discrete bodies and recognize new contacts automatically as the simulation progresses [14]. The most representative DEM methods are the universal distinct element code (UDEC for two dimensions and 3DEC for three dimensions) [15], the particle flow code (PFC^2D^ for two dimensions and PFC^3D^ for three dimensions) [16] and the discontinuous deformation analysis (DDA) method [17]. PFC has been extensively applied to study the brittle fracturing process of rock or rock-like materials in laboratory scale, e.g., compression tests [18,19,20,21,22], both tension and compression tests [23], shear tests on rough discontinuities [24,25,26], and cracking process with pre-existing flaws [27,28,29]. UDEC is another popular DEM code that has been used to simulate the fracturing features in laboratory scale. The grain based models can be built with Voronoi approach in the code and the numerical results can also capture the general features observed in real experiments [30,31,32]. The DDA method is an implicit DEM to simulate the dynamics, kinematics and elastic deformability of a system contacting rock blocks [17,33]. Researchers studied the solid fracturing process with either interface debonding similar to UDEC-Voronooi approach [34] or block fragment into sub-blocks [35]. Some attempts have also been made to combine the DEM with FEM, in which if the fracture criterion within the intact rock (represented by FEM) is met, a crack (represented by DEM) is initiated [36]. The representative codes for this group of approaches include ELFEN [37] and Y-Geo [38,39]. The fracturing process of rock or rock-like materials under various stress conditions have also been studied based on these approaches, e.g., ELFEN in [40,41,42], Y-Geo in [43,44]. The results indicated that the hybrid continuum and discrete methods can simulate the fracturing process well. If interested in, readers can address a detailed review of DEM method in [33,45,46]. These applications showed that the DEM approach has advantages to simulate the fracturing behavior in heterogeneous rock mass that the inherent microscopic heterogeneity of rock can be represented explicitly [47]. However, this approach is time-consuming, and the micro-scale properties (e.g., particle bond strength, grain size distribution) should be calibrated by abundant numerical trails [19]. Besides, it is also not suitable for large scale rock projects, which would involve millions of particles [47].

The continuum approaches, e.g., finite element method (FEM) and finite difference method (FDM), have also been applied to study the heterogeneous material but should be combined with the statistical model and damage model [47]. One of the most representative codes is the FEM based RFPA that was developed by [48], which can simulate the non-linear deformation, strain softening and failure process of quasi-brittle materials by introducing an ideal brittle constitutive law and heterogeneity of microstructural rock property for the local material. In the model, the elemental strength and elastic modulus were described by Weibull distribution function to represent the microstructural heterogeneity. The codes have further extensions and wide applications after being developed. Brazilian indirect tension tests, compression tests with and without confinement as well as microscopic events based on RFPA were studied and verified with the experimental observation [49,50,51]. The influence of heterogeneity on the rock crack propagation was numerical studied by [52], which showed that the crack propagation path is smooth and persistent in homogeneous specimen, comparing with rough and non-persistent in heterogeneous specimen. Liu et al. (2004) suggested the variation in micro-fracture distribution or fracture roughness serve as an indicator of the degree of the specimen heterogeneity after comparison of crack initiation stress, crack distribution and fracture surface between homogeneous and heterogeneous specimens based on RFPA based R-T^2D^ [12]. A three dimensional codes-RFPA^3D^ was also developed and used to study the failure process [53]. Zhu et al. (2015) used RFPA to study fracturing process of rock mass around underground excavations [54]. Tang et al. (2001) studied the crack process of brittle rock with existing flaws based on the code [55]. The code was also extended to hydro-mechanical problems introduced with flow module [56,57]. A digital-image-based (DIB) finite element approach was developed based on the numerical code rock failure process analysis (RFPA) to characterize micro-scale rock heterogeneity, and to understand the impact of micro-scale rock heterogeneity on the macro-scale hydromechanical response of rocks [58]. The loading rate is also considered to study both the dynamic failure [59] and time-dependent rheological behavior of heterogeneous brittle rocks [60]. 

Although RFPA code can simulate realistic failure process under various loading conditions and has a number of satisfactory applications, as stated by [47], it still has some limitations, e.g., the confinement dependent mechanical behavior and the deformation response are absent in the code. In addition to the FEM based model, a FDM based local degradation approach coupled with statistic model has also been developed by [61,62]. In this approach, Weibull distribution function was used to present the elemental heterogeneity similar to RFPA and innovatively a degradation index was introduced to incorporate the confinement effects. The approach was successfully applied to failure process of rock specimens in laboratory experiment [63] and some rock projects in engineering scale [64]. The results showed that the confinement effect can be well reflected in this approach, e.g., stress-strain relations that evolve from brittle to ductile as well as fracture patterns that evolve from axial splitting to shear to dilatancy as confinement increases [63]. Some other continuum numerical approaches have also been developed for the heterogeneous material such as smooth-particle hydrodynamics (SPH) [65], cellular automaton [66,67], and lattice models [68,69].

The existing approaches for progressive failure of material have been briefly reviewed above, and in the following part the approach used in this study is presented.

### 2.2. The Approach Used in this Study

**Figure 1:**
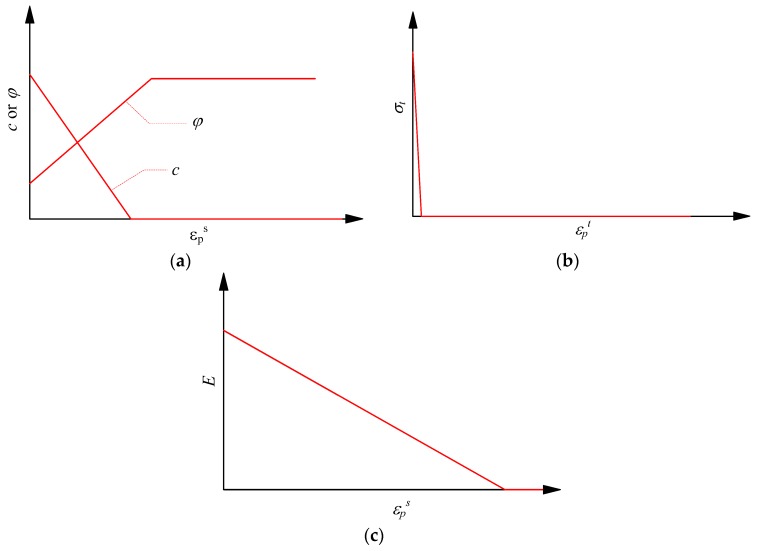
The implemented CWFS-TL degradation strength model as well as elastic modulus degradation model: (**a**) cohesion weakening and friction strengthening; (**b**) tensile strength loss; and (**c**) elastic modulus weakening.

A degradation strength model was proposed mainly based on back analysis of the brittle spalling failure occurring in high stressed Mine by tunnel located in southeastern Manitoba, Canada [7,70]. In this named CWFS strength model, the damage process of brittle rocks indicated cohesion weakening and friction strengthening. Guo et al. (2013) verified the model experimentally based on a series of cyclic loading-unloading compression tests and specially emphasized the tension strength loss as failure process, which is extended as CWFS-TL strength model [71]. This model has been used to predict the spalling shape and mechanism during the excavation at highly stressed rocks, which agree well with the field investigation [72], and also simulate the progressive cracking process of rock specimens with pre-existing, smooth and undulant joint under compression [73,74,75]. In this study, the Mohr-Coulomb failure criterion with a tensional cut-off is used as damage threshold of individual element, and the CWFS-TL strength model is implemented to represent the elemental shear and tensile strength parameters variation during the failure process (Figure 1). It can be seen that the cohesion is weakened linearly to zero and kept as zero, while friction is strengthened linearly to some value and keep constant as the plastic shear strain increases, as shown in Figure 1a. Besides, the elemental tensile strength is lost as soon as tensile failure occurs, as shown in Figure 1b. The elastic modulus is also weakened linearly to zero as the plastic shear strain increases and kept as zero in the residual stage as shown in Figure 1c. These evolution processes of strength parameters can be supported by the experimental results reported in [71]. The relations can also be expressed as Equations (1)–(3), in which the shear strength and elastic modulus are functions of plastic shear strain, and the tensile strength is the function of plastic tensile strain.

(1) $$\tau =\sigma_{n}\tan\left(\varphi_{0}\left(\varepsilon_{p}^{s}\right)\right)+c_{0}\left(\varepsilon_{p}^{s}\right)$$

(2) $$\sigma_{t}=\sigma_{t0}\left(\varepsilon_{p}^{t}\right)$$

(3) $$E=E_{0}\left(\varepsilon_{p}^{s}\right)$$

where *τ* denotes the shear strength; *σ_n_* denotes the normal stress at failure; *σ_t_* denotes tensile strength; *E* denotes elastic modulus; $\varphi =\varphi_{0}\left(\varepsilon_{p}^{s}\right)$, $c=c_{0}\left(\varepsilon_{p}^{s}\right)$ and $E=E_{0}\left(\varepsilon_{p}^{s}\right)$ denotes friction angle, cohesion and elastic, respectively, which are all functions of plastic shear strain; $\sigma_{t0}\left(\varepsilon_{p}^{t}\right)$ denotes that tensile strength is the function of plastic tensile strain; *c*_0_, *ϕ*_0_, *σ_t_*_0_ and *E*_0_, respectively, denote cohesion, friction angle, tensile strength and elastic modulus of element with no damage.

**Figure 2:**
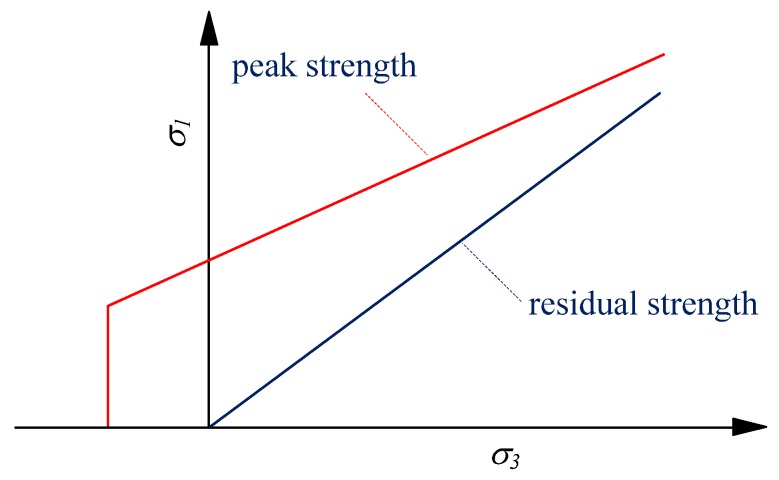
Envelope of peak and residual strength of the elements.

The envelopes of peak and residual strength are shown in Figure 2. It can be seen that from peak to residual, the tensile strength and cohesion drops to zero, while the frictional strength is mobilized and increased to a residual value. The differences between presented strength envelopes and other ones are obvious, e.g., a constant friction angle was used in [48] that cannot incorporate the confinement effects as mentioned above. Alternatively, a new degradation index without fundamental physical meaning, was introduced to reflect the confinement effects in [62,63].

**Figure 3:**
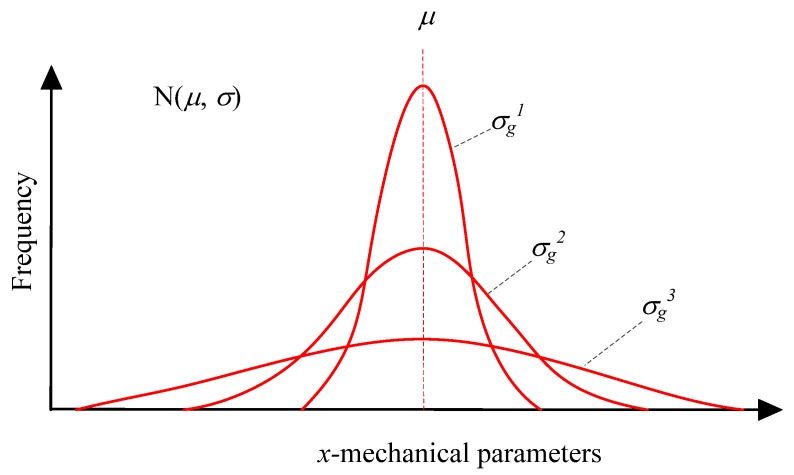
The normal distribution of the mechanical parameters.

As mentioned above, the behaviors of rocks or rock-like materials always exhibit a large amount of experimental scatter. At the same time the various observed phenomena within one given class of problems show very strong similarities, indicating that their randomness is highly affected by a strong deterministic background [12]. The rocks and rock-like materials heterogeneity of elemental properties is described by Weibull distribution function in some previous codes, e.g., RFPA in [48] and the local degradation approach developed by [62]. However, experiments reported in the publications indicate that the strength and deformation parameters of rocks or minerals always follow Gaussian distribution function, e.g., uniaxial compressive strength of Marinenda sandstone [10], granite [11] and the indentation modulus of minerals including quartz, feldspar and biotite [43]. In this study, the Gaussian distribution is used to depict the elemental heterogeneity (Equation (4) and Figure 3).

(4) $$f(x)=\frac{1}{\sqrt{2\pi }\sigma_{g}}e^{-\frac{{\left(x-\mu \right)}^{2}}{2{\sigma_{g}}^{2}}}$$

where *μ* denotes the mean value of *x*, and *σ_g_* denotes the standard deviation that describes the scatter of *x*. The subscript *g* differentiates the Gaussian parameters *σ_g_* with other idiomatic usages, e.g., *σ* of stress or strength. The Gaussian distribution function is also named normal distribution and abbreviated as N (*μ*, *σ_g_*).

In Equation (4), *x* is the variable that follows Gaussian distribution, and would be used to represent the elemental tensile strength, cohesion, friction angle and elastic modulus in the material model. Figure 3 shows three Gaussian distributions function curves with various *σ_g_*, i.e., ${\sigma_{g}}^{1}<{\sigma_{g}}^{2}<{\sigma_{g}}^{3}$. It can be seen that the bigger *σ_g_* indicates flatter curve and more scattered *x*. Although *σ_g_* can reflect the scatter degree of one number set, it cannot give a suitable indicator for more than two number sets which have different mean values or dimensions. The ratio of *σ_g_* to *μ*, termed the coefficient of variance, is standardized of *σ_g_* and always introduced to depict the scatter of several number sets. The coefficient of variance would be used to depict the heterogeneity degree of material properties mentioned above, which is denoted by *h_cv_* in this study. The existing tests showed that Poisson’s ratio has little effect on the modeling results [76], and hence a constant Poisson’s ratio is used for all the elements for simplicity. 

In this study, the FDM based program, Fast Lagrangian Analysis of Continua in 3 Dimensions (FLAC^3D^), is used associated with the plastic strain dependent CWFS-TL strength model and statistical model stated above. In FLAC^3D^, materials are represented by polyhedral elements within a three-dimensional grid that is adjusted by the user to fit the shape of the object to be modeled. Each element behaves according to a prescribed linear or nonlinear stress/strain law in response to applied forces or boundary restraints. The material can yield and flow, and the grid can deform (in large-strain mode) and move with the material that is represented [77]. FLAC^3D^ embodies a number of basic constitutive models for use in the analysis of the mechanical behavior of geomaterials and user-defined constitutive models can also be implemented based on built-in programming language. In this way, FLAC^3D^ has been adopted to study the progressive failure of rock materials with various joints [73,74,75] and deep excavation [72] associated with the presented plastic strain dependent CWFS-TL strength model. Furthermore, the elemental heterogeneity described by Gaussian distribution function would also be incorporated in the program based on the built in programing language in this study.

## 3. Description of the Heterogeneous Material Models and the Uniaxial Compression Tests

### 3.1. The Heterogeneous Material Models

**Figure 4:**
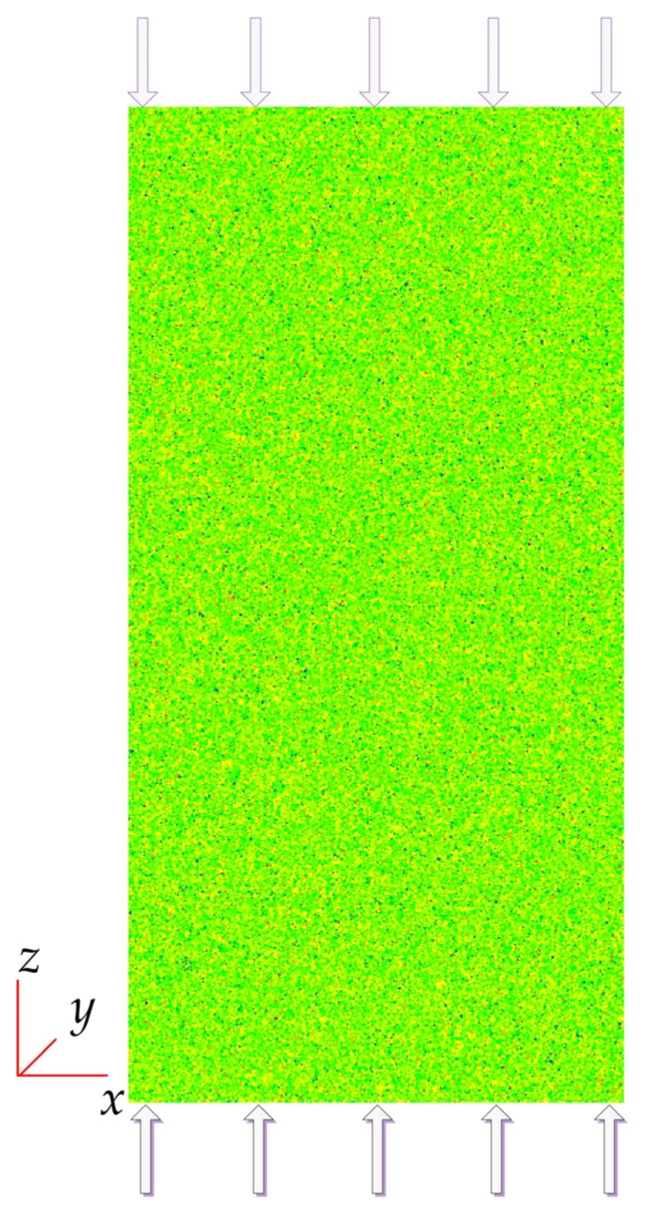
The heterogeneous models in FLAC^3D^.

The size of model in direction of *x*, *y* and *z* is 76 mm × 1 mm × 152 mm, represented by 152 × 1 × 304 = 46,208 uniformly distributed wedge elements in mesh (Figure 4). The axes of *x*, *y* and *z* indicate the width, thickness and height of the model, respectively. Different elements are represented by various colors in Figure 4. 

As stated above, the mechanical parameters needed in this study can be divided into four groups: first, parameters that determine the elemental deformation features (i.e., elastic modulus *E*, and Poisson’s ratio *v*); second, parameters that determine the elemental failure threshold (i.e., the cohesion *c*_0_, friction angle *ϕ*_0_, and tension *τ*_0_); third, parameters that determine the property heterogeneity (i.e., the mean value of above parameters *μ* and the heterogeneity degree *h_cv_*); and fourth, formulation that determines the evolutional process of the above mechanical parameters as plastic strain increases. The mean parameters or formulation of first, second and fourth groups are listed in Table 1, associated with the various heterogeneity degree parameters *h_cv_*. The mean parameters of elastic modulus *E*_0_, cohesion *c*_0_, friction angle *ϕ*_0_, and tension *τ*_0_ at peak are 60 GPa, 50 MPa, 38° and 20 MPa, respectively. It can be seen that the ratio of mean uniaxial compressive strength (*σ_c_*_0_), i.e., 205 MPa, to mean tensile strength is about 10. The corresponding parameters of individual elemental are following the Gaussian distribution function N (*μ*, *μh_cv_*) in which *μ* represents the mean value. When the local stress of an individual element reaches its peak strength threshold, its parameters will change following the formulations listed in the third column shown in Table 1, and when the plastic strains reach critical values, the residual deformation and strength parameters will take effect. For simplicity, the critical plastic shear strain 0.1% is adopted to determine the softening to residual at the post-peak stage for the elastic modulus and shear strength parameters, while a much smaller plastic tensile strain 0.01% is adopted to determine such transform for tensile strength, which are roughly based on the experiments of marbles in [71]. All the residual tensile strength, cohesion and elastic modulus are weakened to zero, while the residual friction angle is mobilized to 40° according to the experiments in [71] and the rock slip tests summarized in [78]. As stated above, a constant Poisson’s ratio of 0.25 is adopted.

**Figure 5:**
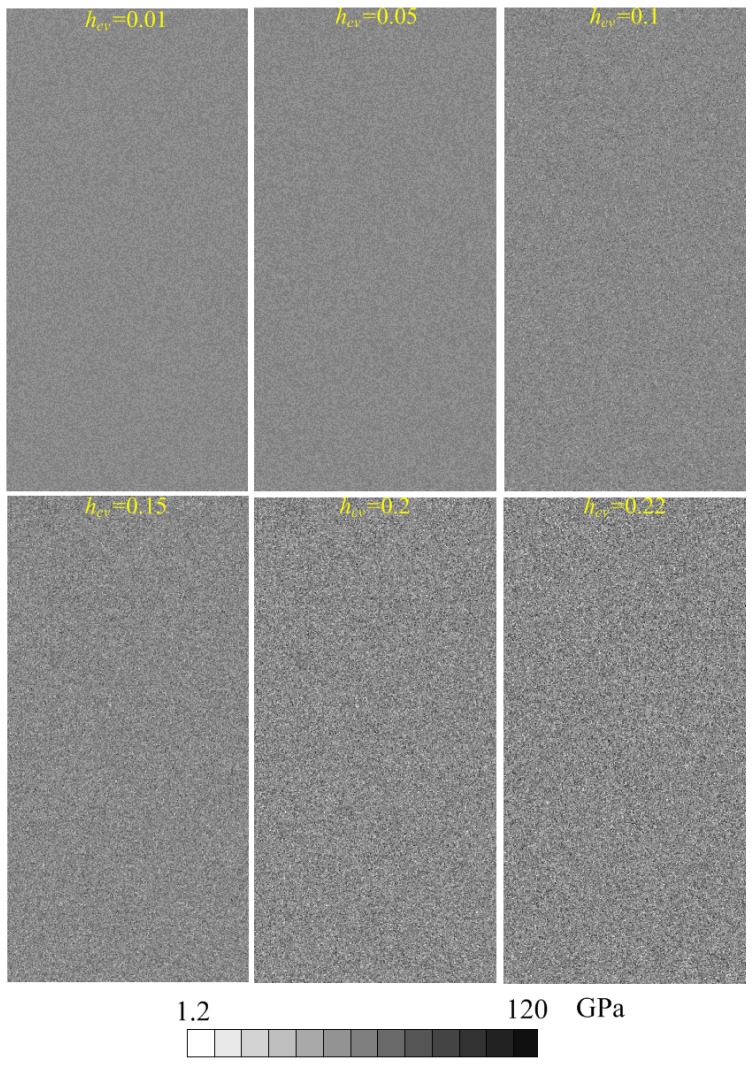
The heterogeneous models with inhomogeneous mechanical parameters (elastic modulus as an example) following Gaussian distribution function.

**Figure 6:**
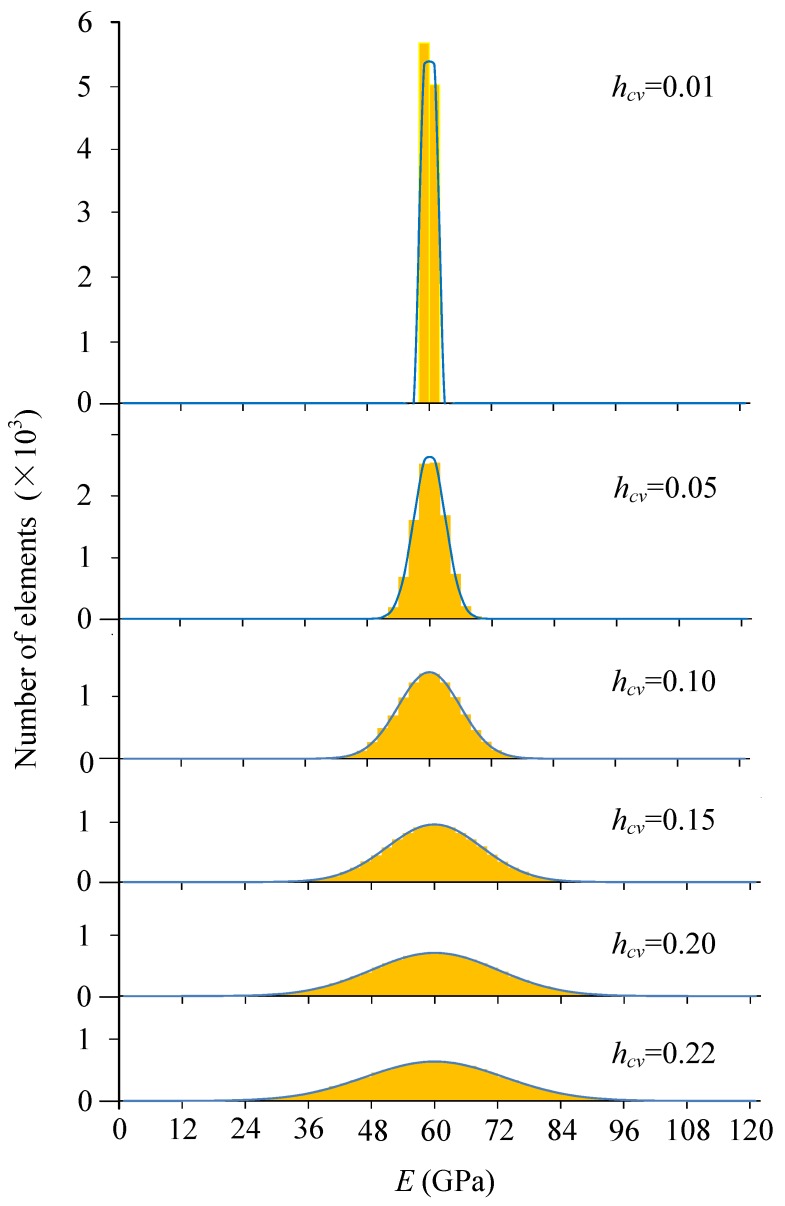
The histogram of elastic modulus with different heterogeneity index *h_cv_*.

Associated with the mean parameters, the heterogeneity parameters of *h_cv_* were adopted for six levels to build the heterogeneous material model, i.e., 0.01, 0.05, 0.10, 0.15, 0.20 and 0.22. As an example, the elemental elastic moduli of the models are shown in Figure 5. The scales of grey color shades indicate magnitude of the parameters. Figure 6 shows the histogram of elastic modulus distributions of the models given in Figure 5. It can be seen that as the *h_cv_* increases, the scatter of elastic modulus increases and the materials tend to be more heterogeneous. 

### 3.2. Description of the Numerical Uniaxial Compression Tests

The numerical uniaxial compression tests are conducted with FISH code in FLAC^3D^ on the model shown in Figure 4. The axial load (*z* direction) is applied to the ends of the specimen model, with a strain rate increment of 10^−6^ according to suggested method by ISRM [79]. The displacement is fixed in *y* direction during the compression tests, and free in *x* direction. Therefore, the model is actually in plane strain state and without confinement. The overall as well as local stress and strain data are recorded during the tests. Besides, similar to RFPA [48], it is assumed that the number of acoustic emission (AE) events is equal to failed elements and also recorded in each step. 

## 4. The Stress and Fracturing Process of Heterogeneous Material under Uniaxial Compression

In this section, in the first part the material with one typical heterogeneity degree (*h*_cv_ = 0.2) is presented in detail as an example to study the stress and fracturing process of the heterogeneous material model under uniaxial compression, and in the second part the influence of heterogeneity is discussed.

### 4.1. The Failure Process

**Figure 7:**
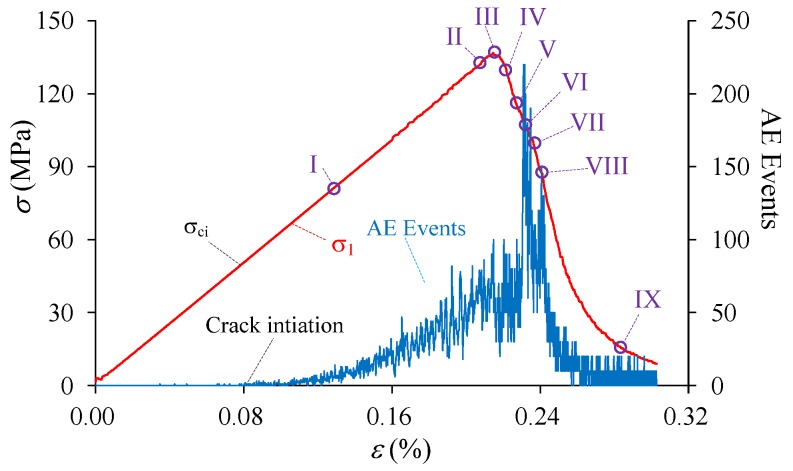
The stress-strain relation and AE events of the heterogeneous model under uniaxial compression (*h_cv_* = 0.2).

The stress-strain curve as well as crack growth (AE events) of the heterogeneous material during compression is shown in Figure 7. It is shown that the curve presents typical heterogeneous features that have a non-linear deformation stage (I–III) in the pre-peak stage and an obvious strain-softening behavior in the post-peak stage (III–IX), which is in good agreement with the results in experiments of uniaxial compression test on brittle rocks or rock-like materials, e.g., marble [80], granite and sandstone [81], and concrete [82]. The uniaxial compressive strength, i.e., 136.9 MPa for this case of *h_cv_* = 0.20, is weakened compared with the mean strength of the element due to the existence of heterogeneity. The cracking process can be numerically analyzed through the number of acoustic emission (AE or damage) events accompanied with the stress-strain curve shown in Figure 7, on the basis of which the crack initiation and the corresponding stress threshold (*σ*_ci_) can be identified. It is shown that the crack initiation stress is 49.78 MPa, about 0.36 of the peak stress in this case. After crack initiation, the number of AE events increases steadily before Point I, and after that begins to increase non-linearly until the eventual failure occurs. However, most AE events, even the highest one, occur in the post-failure stage instead of at the peak stress. This observation is in accordance with the numerical studies including RFPA [51], the FDM-based approach [63], SPH model [65] as well as the experimental results of sandstone and granite [83]. In these studies, researchers found that under uniaxial and low confining pressure, the highest acoustic events or the macroscopic fracture plane occurred after peak stress.

**Figure 8:**
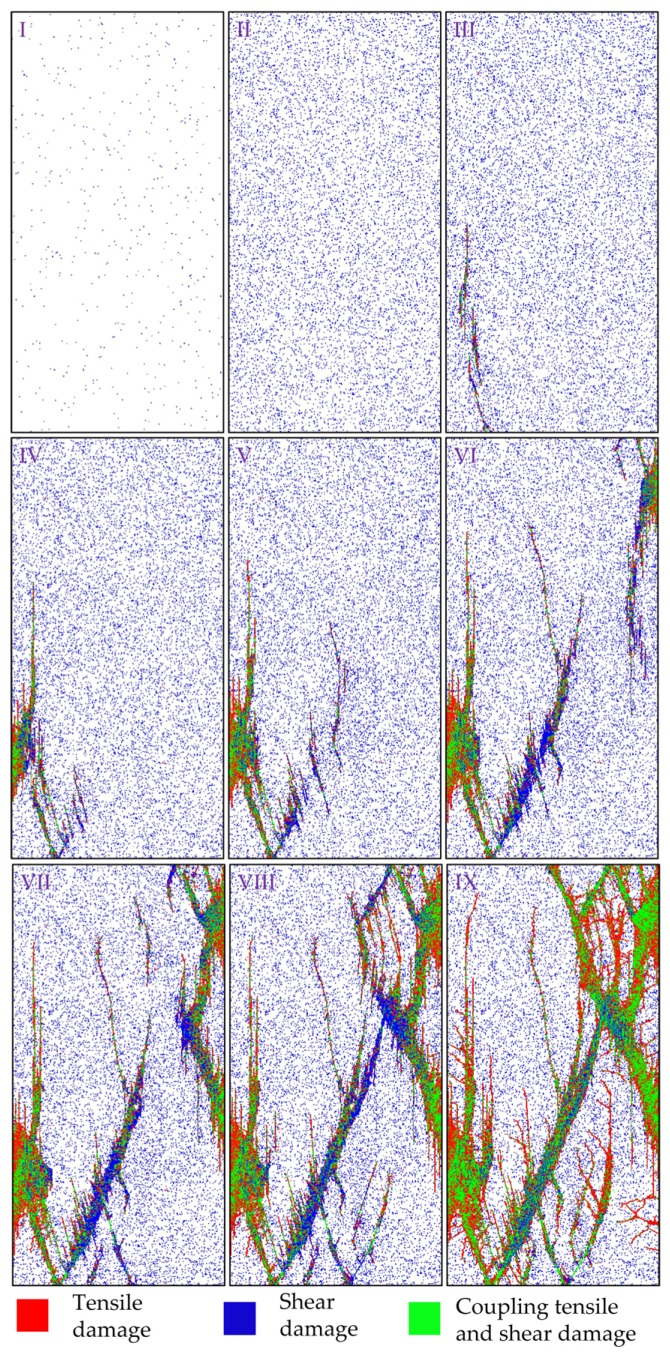
The fracturing process of the heterogeneous model under uniaxial compression (*h_cv_* = 0.2).

**Figure 9:**
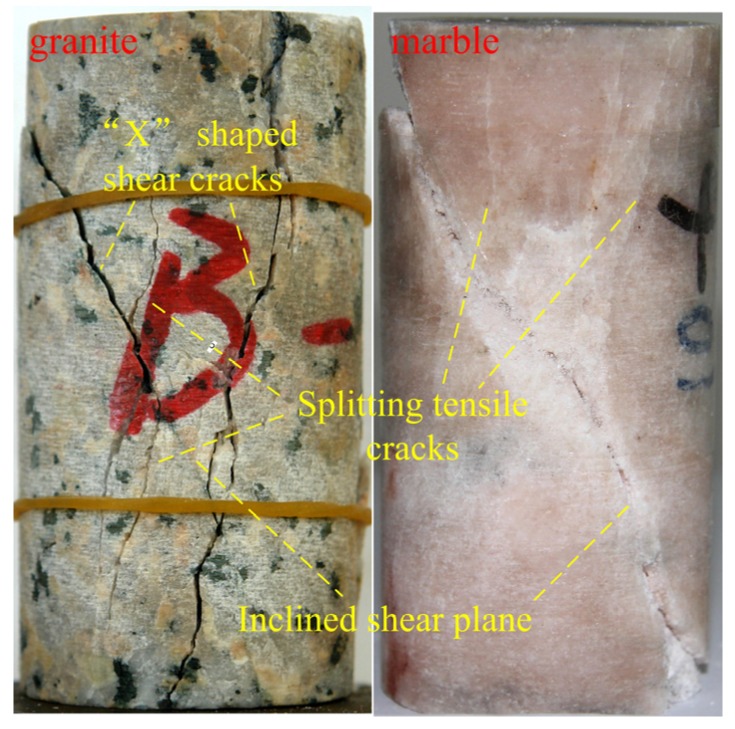
The failure mode of granite and marble under uniaxial compression.

The fracturing process of the heterogeneous model corresponding the Points I–IX at different stages in Figure 7 is presented in Figure 8. In the figure, the red color represents tensile damaged (or failed) elements, blue color represents the shear damaged elements, and green color represents the coupling tensile and shear damaged elements. At the linear deformation stage (Point I in Figure 7 and Figure 8), the damage events are randomly distributed in the specimen because of the elemental heterogeneity. As the external loading increases, the randomly distributed damaged elements become denser and tend to get clustered in some location, and the non-linear deformation occurs in the pre-peak stage (Point II in Figure 7 and Figure 8). Clear localized damaged zones initiate and propagate nearly parallel with the axial loading direction in the left bottom position of the model at peak stress (Point III in Figure 7 and Figure 8). After the peak stress, there is a big stress drop and strain-softening phenomenon is observed in the stress-strain curve (Points IV–VIII in Figure 7). Some new damaged macro cracks are formed with en echelon arrangement left bottom part of specimen (Points IV and V in Figure 8). These cracks coalesce as a shear zone along an inclined plane cutting through left half part of the specimen, and some new macro cracks initiate in the right top position as well as the highest AE events occur in this stage (Point VI in Figure 7 and Figure 8). It should be pointed out that several tensile cracks also initiate and tend to propagate sub-parallel with the loading direction at tips of the damaged zone at the same time. The inclined shear plane and the new initiated cracks grow gradually (Point VII in Figure 8), coalesce and form an inclined shear plane that cuts through the specimen (Point VIII in Figure 8). At the last point (Point IX in Figure 7 and Figure 8), the model reaches residual state and eventual failure mode can be observed, i.e., inclined “X” shaped shear planes as well as some splitting tensile fractures. Such a failure mode, i.e., combination of axial splitting and inclined failure surfaces, was always observed and has been manifested as a common mode for brittle rocks failure under uniaxial compression tests, e.g., the failure types shown in Figure 9 including a marble sample with uniaxial compressive strength of about 78 MPa and a granite sample with uniaxial compressive strength of about 89 MPa. 

From above, the conducted approach in this paper is verified that the features of stress-strain relation, AE events and failure modes observed in laboratory tests or the simulation performed by others can all be captured in this numerical simulation.

**Figure 10:**
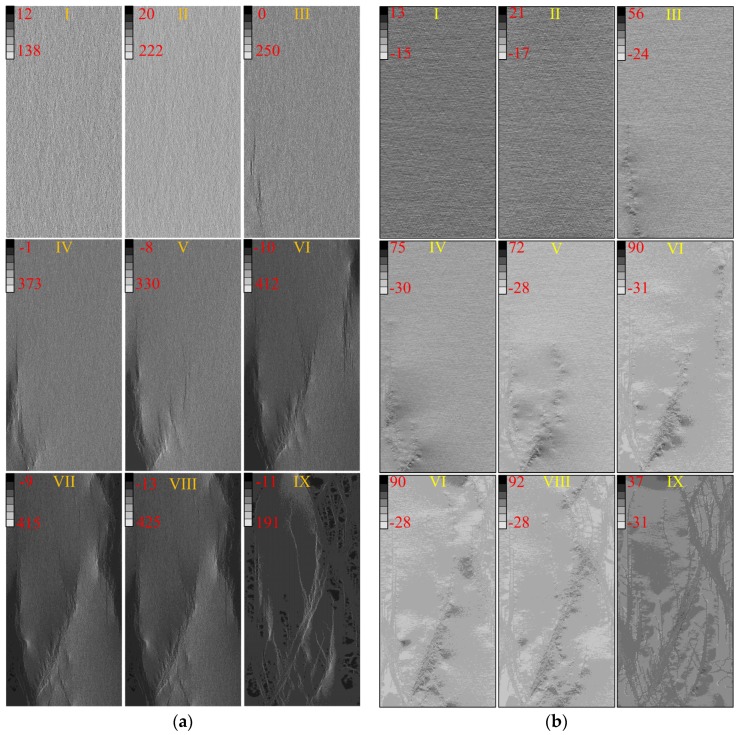
The maximum and minimum principal stress contour of the heterogeneous model under uniaxial compression (*h_cv_* = 0.2): (**a**) *σ*_1_; (**b**) *σ*_3_.

As mentioned in the Introduction Section, the elemental stress was disturbed due to the material heterogeneity in the linear deformation stage under external loads. As some weak elements were damaged due to the inhomogeneous stress, the damage events and stress redistribution influence each other mutually throughout the whole failure process. The maximum (*σ*_1_) and minimum principal stress (*σ*_3_) contours corresponding to Points I–IX are shown in Figure 10. The grey scale in Figure 10 represents the value of *σ*_1_ in Figure 10a and *σ*_3_ in Figure 10b respectively. The positive value represents the compressive stress while the negative value represents tensile stress in the figure. It can be seen from Figure 10a that distribution of *σ*_1_ seems uniform in the specimen scale but non-uniform in the element scale, and increase gradually from Point I to Point II. At the peak stress, i.e., Point III, an obvious stress concentration zone is observed in the left bottom accompanied with the initiated fracture zone shown in Figure 8. In the strain-softening stage, i.e., from Point IV to Point VIII, the highest elemental *σ*_1_ increases while the lowest elemental *σ*_1_ decreases gradually, which indicates that the stress difference between elements become greater and stress concentration gets higher as the development of fracturing process. Besides, it is found that the relatively high elemental *σ*_1_ (brighter color) always appear near the boundary of the shear failure zones if comparing Figure 8 and Figure 10. At the residual stage (Point IX), the inclined slip surfaces are formed and the specimen is yield. Therefore the highest elemental *σ*_1_ decreases and lowest elemental *σ*_1_ increase, which indicates the stress distributes more uniformly than Points III‒VIII.

On the other hand, distribution of *σ*_3_ seems also generally uniform in specimen scale but highly non-uniform in element scale at linear deformation stage (Points I and II in Figure 10b). The negative values of *σ*_3_ indicate the tensile stresses appear in some elements and the lowest elemental *σ*_3_ increases gradually in the pre-peak stage. At peak stress (Point III), the stress near damage zones was disturbed strongly, which has similar distribution features with that at the strain softening stage (Points IV‒VIII). It is found that the concentrated tensile stresses always appear near the boundary, especially at the tips, of the failure zone, where a relatively great difference of mechanical properties exists between the damaged and undamaged elements. At the residual stage (Point IX), the specimen is yield along the inclined shear failure surfaces and the elemental *σ*_3_ decreases compared with that in the strain-softening stage.

Although the process of elemental *σ*_1_ and *σ*_3_ distribution can provide very valuable information to understand the progressive failure of the heterogeneous material in the presence of external loads, both the overall and elemental stress condition should be considered together if analyzing the stress concentration level of the individual element. Guo and Qi (2015) [75] used an index of stress concentration factor (*SCF*) that incorporated both the overall and local principal stress difference to denote the degree of heterogeneous stress (Equation (5)).

(5) $$SCF=\frac{\sigma_{1}-\sigma_{3}}{{\left(\sigma_{1}-\sigma_{3}\right)}_{o}}$$

where *σ*_1_ − *σ*_3_ is the localized principal stress difference of elements in the material; and (*σ*_1_ − *σ*_3_)*_o_* is the overall principal stress difference applied onto the material.

**Figure 11:**
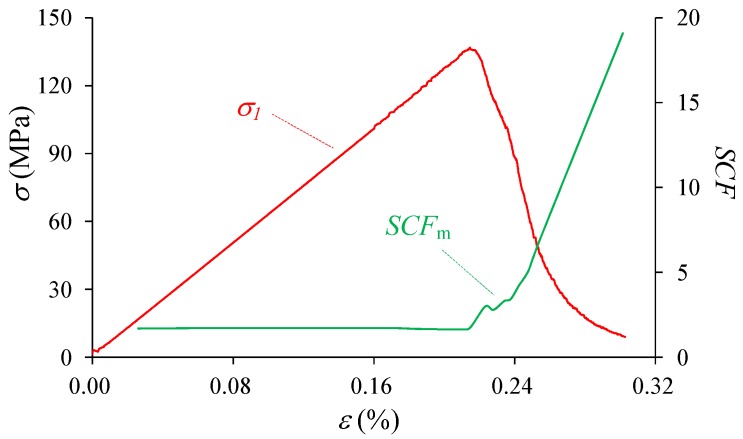
The maximum stress concentration factor (*SCF_m_*) of the heterogeneous model under uniaxial compression (*h*_cv_ = 0.2).

The *SCF* is a normalized index that considers the ratio of local stress and overall stress, and hence can reflect the localized stress concentration well. In this study, the *SCF* index is applied and the maximum *SCF* of all the elements, i.e., *SCF_m_*, during the failure process is presented in Figure 11. It can be seen that the localized stress starts to get concentrated at the initiation of external loads, and *SCF_m_* keeps about 1.69 during the elastic deformation. At and after the peak stress, *SCF_m_* increases sharply as the failure develops, mainly resulting from the increased elemental stress and decreased overall stress.

Taking the material with *h*_cv_ = 0.2 under uniaxial compression as an example, the stress-strain relation, AE events, progressive crack features, as well as the elemental *σ*_1_, *σ*_3_ and stress concentration condition during the failure process are analyzed in this part. In the next part, the influence of heterogeneity on the fracturing and stress process would be studied considering various *h*_cv_.

### 4.2. The Influence of Material Heterogeneity

**Figure 12:**
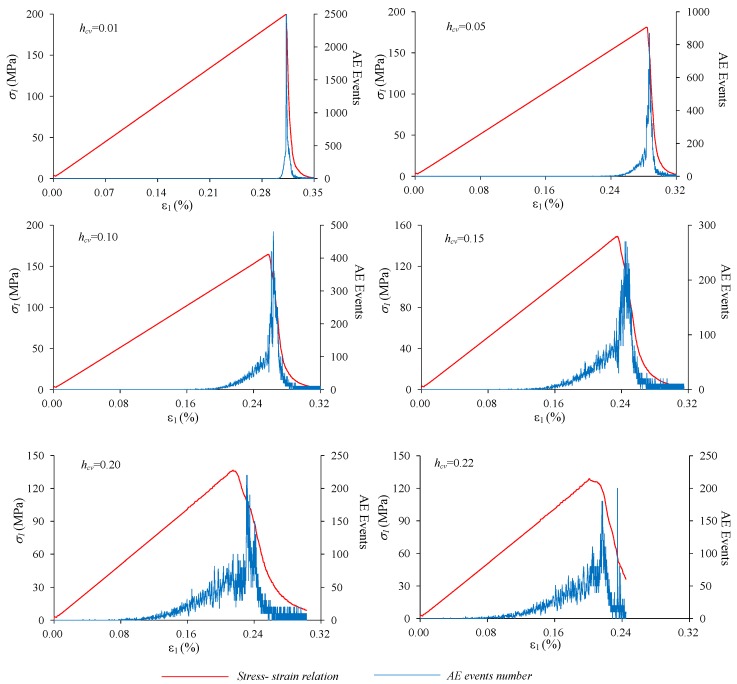
The stress-strain relations and AE events process of material with various heterogeneity index *h*_cv_.

**Figure 13:**
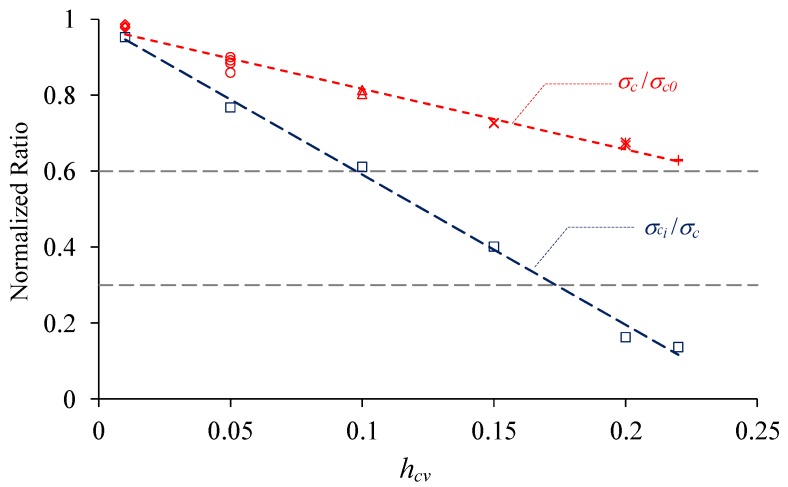
The normalized crack initiation stress and peak stress of material with various heterogeneity index *h*_cv_.

As described in Section 2, uniaxial compression tests are numerically conducted on materials with six levels of *h*_cv_, i.e., 0.01, 0.05, 0.10, 0.15, 0.20 and 0.22. The stress-strain relations as well as the AE events process of these materials under uniaxial compression are shown in Figure 12. It can be seen that the peak stress decreases as *h*_cv_ increases and the strength of these heterogeneous materials are all weakened compared with the mean strength of the elements. If the strengths are normalized by the mean strength, a linear relation would be observed between the strength and heterogeneity index *h*_cv_ of the material (red signals and dashed lines in Figure 13). 

The brittleness of stress-strain curve decreases as the heterogeneity index h_cv_ increases. For the relatively homogeneous material, e.g., *h*_cv_ = 0.01, the AE events are rarely observed until nucleates abruptly just before the peak stress, and then the macro fracture are formed with sharp stress drop. A large amount of elements (about 27,000) were damaged in only a short period (Δε = 0.03%). Conversely, for the relatively heterogeneous material, e.g., *h*_cv_ = 0.22, AE events initiate at the early elastic deformation stage and increase with the external loads resulting to about half AE events occurring before peak stress. Although the highest AE events occur in the post peak stages, AE events seem more disperse than those in the relatively homogeneous material, about 21,000 elements are damaged in a long period of Δε = 0.18%. Another interesting observation is that the position of the highest AE events locates in the post-failure stage but that position moves towards the peak stress for the material with lower heterogeneity degree, from which it can be indicated that the delay of the highest AE events, or macro rupture, are mainly resulting from material heterogeneity.

Based on the AE events process shown in Figure 12, the crack initiation stress (*σ_ci_*) can be identified and presented in Figure 13 as blue squares and dashed line after normalized by the peak stress (*σ_c_*). The results show that the ratio *σ_ci_*/*σ_c_* decreases linearly as heterogeneity index *h*_cv_ increases. It is indicated that the crack initiation stress is nearly equal to the peak stress for the very homogeneous material, and the crack may nucleate at a very low stress compared to the peak stress for the relatively heterogeneous material. Furthermore, if the range of this ratio for rocks is considered, i.e., 0.3–0.6 as mentioned in the Introduction Section, it is suggested that the values range between 0.12 and 0.21 for *h*_cv_ are appropriated to represent the property heterogeneity for rock materials (Figure 13). 

**Figure 14:**
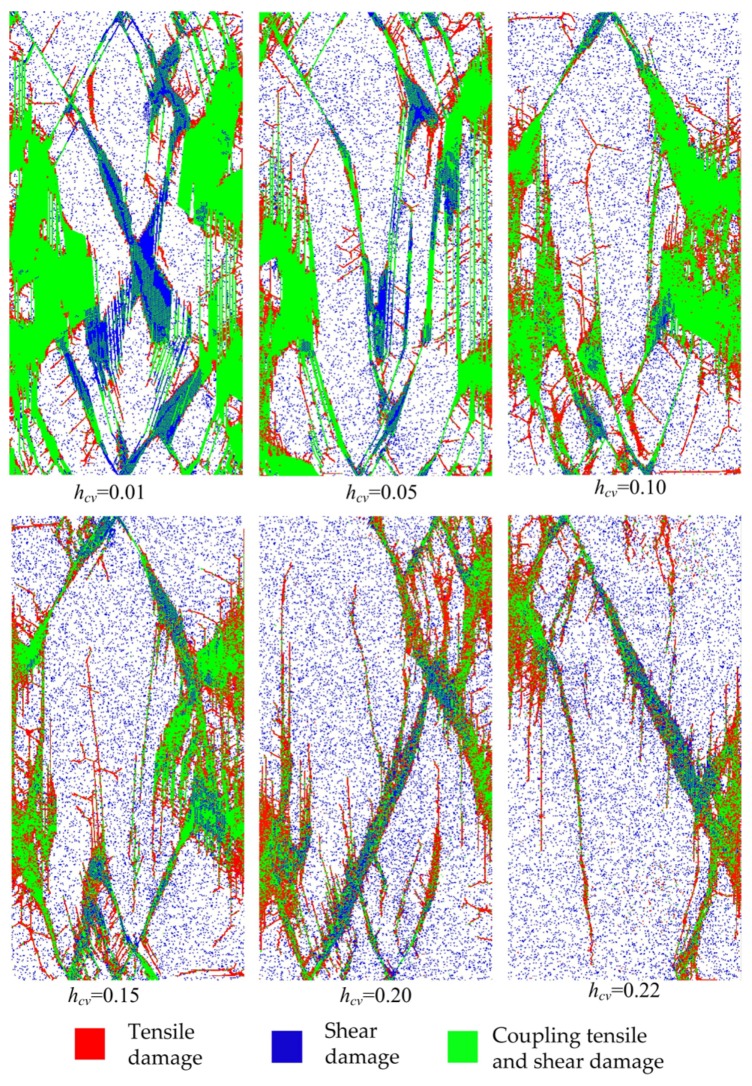
The ultimate failure modes of material with various heterogeneity index *h*_cv_.

The eventual failure modes are shown in Figure 14, from which the similar conclusion can be reached that *h*_cv_ between 0.12 and 0.21 is suitable for rocks. Compared with the common brittle rock failure under uniaxial compression in experiments, i.e., the splitting tensile fractures sub-parallel with axial loads and inclined shear plane, the fracture forms in an unlikely way for materials with *h*_cv_ = 0.01, 0.05 and 0.1, while the failure modes of materials with *h*_cv_ = 0.15, 0.20 and 0.22 are more likely for the rock failure.

**Figure 15:**
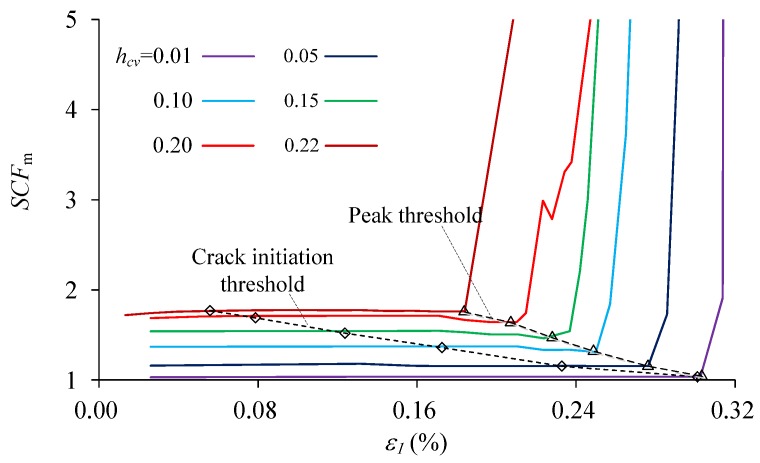
The maximum stress concentration factor (*SCF*_m_) during the process of uniaxial compression of the materials with various heterogeneity index *h*_cv_.

**Figure 16:**
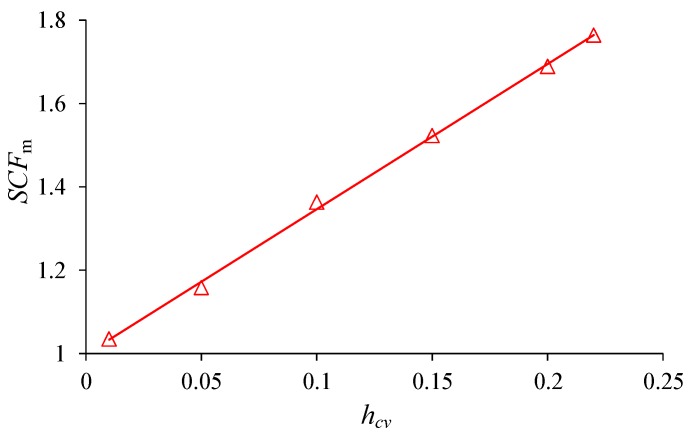
The stress concentration index *SCF*_m_ at the elastic deformation stage of material with various heterogeneity index *h*_cv_.

The maximum elemental stress concentration factor (*SCF_m_*) of the material with various heterogeneity index *h*_cv_ during the uniaxial compression can be obtained in Figure 15. It is shown that *SCF_m_* have no obvious fluctuation before the peak stress, even before and after crack initiates. When the macro failure zone nucleates around peak stress, *SCF_m_* begins to increase sharply in the post peak stage. The mean *SCF_m_* in the elastic deformation stage has a perfect linear relation with *h*_cv_ (Figure 16). It is indicated that the magnitude of stress concentration are mainly dependent on the heterogeneity index. 

## 5. Discussion

The mechanical behaviors of materials are heterogeneity dominant and it is of great importance to find a suitable index that can represent the material heterogeneity. As mentioned in the Introduction Section, the Weibull distribution function was adopted to depict the mechanical parameters of the elements in some researches, e.g., [48,63]. The shape parameter *m* in the function defines the degree of material homogeneity and is referred to as the homogeneity index. Materials with higher *m* values are more homogeneous, whereas those with lower *m* values are more heterogeneous. Although the qualitative relation is obvious and known well, there are no quantitative relation between *m* and the mechanical parameters of the materials. In this study, the Gaussian distribution function is adopted to describe the material heterogeneity and the *h*_cv_ (coefficient of variance) in the function is used to define the heterogeneity degree. The material is completely homogeneous for *h*_cv_ = 0, and the heterogeneity degree increases with *h*_cv_. Furthermore, a linear quantitative relation is found between *h*_cv_ and some critical thresholds or stress concentration factors, i.e., peak stress or uniaxial compressive strength (*σ_c_*), ratio of crack initiation stress to peak stress (*σ_ci_*/*σ_c_*) and mean *SCF_m_* in elastic deformation stage (Figure 13 and Figure 15). 

Researches have shown that various materials have different heterogeneity degrees. If Weibull distribution function is adopted, McClintock and Aegon (1966) suggested that values of 2–6 for *m* for engineering materials [84], and Fang and Harrison (2002) found that values of 2–4 are most appropriate for most rocks based on the failure features of the numerical studies [63]. In this study, the values of 0.12–0.21 are reliably suggested for *h*_cv_ to represent the heterogeneity degrees of the most rocks after considering the range of *σ_ci_*/*σ_c_* as well as the failure modes for rocks. The relatively homogenous materials always have higher *σ_ci_*/*σ_c_* indicating that failure of such materials is violent and difficult to predict. The rock bursts occurring in intact or blocky brittle rocks which are less heterogeneous than jointed rock masses are good examples that always occur for such failure [85]. Inversely, the relatively heterogeneous materials always have lower *σ_ci_*/*σ_c_*, e.g., 0.13 for the jointed granite that are highly heterogeneous rock mass at the ZEDEX tunnel, as mentioned in the Introduction Section [9].

**Figure 17:**
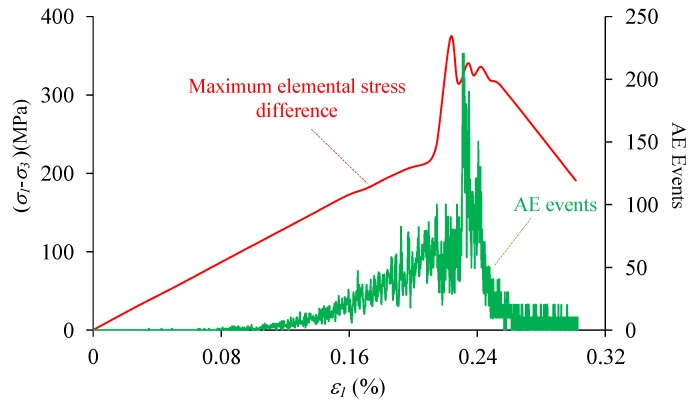
The maximum elemental stress difference and AE events during the uniaxial compression (*h*_cv_ = 0.2).

One interesting observation is that the highest AE events occur in the post peak stage but not before or at peak stress. If the local stress is analyzed with the AE events, it is found that although the overall stress has been dropped down at the highest AE events point, the maximum local stress is well corresponding to the AE events process, especially in the post peak stage (Figure 17). It is indicated that there are proportional relation between the overall and local stress in the linear deformation stage, but after peak the local stress increases sharply as the damages propagate and coalesce, and drops after the macro failure is formed. This phenomenon may give a reasonable explanation for the facts of AE events delay.

## 6. Conclusions

The Gaussian distribution function is adopted to describe the property heterogeneity of materials, in which the coefficient of variance is introduced to define the heterogeneity degree. By implanting a plastic-strain dependent CWFS-TL strength model developed based on the experimental results and back analysis, the FDM approach FLAC^3D^ is used to study the failure process and stress disturbance during the uniaxial compression, as well as the influence of material heterogeneity.

The simulation results indicate the approach can capture the observation in experiments or other numerical studies. Firstly, the stress-strain curve presents typical heterogeneous features that have a non-linear deformation stage in the pre-peak stage and an obvious strain-softening behavior in the post-peak stage. Secondly, the crack initiation, propagation and coalescence are observed and the eventual failure modes are combination of axial splitting tension and inclined shear. Thirdly, the highest AE events occur in the post-failure stage instead of at the peak stress. It is indicated that the peak stress (strength) and crack initiation stress are both heterogeneity dependent. The heterogeneity degree index *h*_cv_ has a perfect linear relation with the normalized *σ_c_* and *σ_ci_*. A range of 1.2–2.1 is suggested for *h*_cv_ to represent the properties of most rocks if considering the threshold of crack initiation stress and failure modes. 

The local maximum and minimum principal stress distribution during compression are analyzed comparing with the failure process. It is found that the stress concentration appears in the undamaged elements at the boundary of failure sites. The stress concentration factor (*SCF*), ratio of local stress difference to overall stress difference, is introduced to represent the local stress concentration condition. It is found that the localized stress starts to get concentrated at the initiation of external loads, and the maximum SCF (*SCF_m_*) keeps nearly unchanged during the elastic deformation but increases sharply as failure develops after peak. It is found that the mean *SCF_m_* in the linear deformation stage is also heterogeneity dependent and has a good linear relation with *h_cv_*. The local stress difference has a significant influence on the damage process and may give a satisfactory explanation to the facts of AE events delay compared with the overall stress-strain curve. 

## Figures and Tables

**Table 1:** The mean mechanical parameters of model elements.

**Plastic Strain Parameters**	$\varepsilon_{p}^{s}=0$	$0<\varepsilon_{p}^{s}\leq 0.1\%$	$\varepsilon_{p}^{s}>0.1\%$
**Elastic Modulus (Gpa)**	60	$60\times \left(1-1000\varepsilon_{p}^{s}\right)$	0
**Poisson’s Ratio**	0.25	0.25	0.25
**Cohesion (MPa)**	50	$50\times \left(1-1000\varepsilon_{p}^{s}\right)$	0
**Friction Angle (°)**	38	$2000\times \varepsilon_{p}^{s}+38$	40
**Tensile Strength (MPa)**	$\varepsilon_{p}^{t}=0$	$0<\varepsilon_{p}^{t}\leq 0.01\%$	$\varepsilon_{p}^{t}>0.01\%$
	20	$20\times \left(1-10000\varepsilon_{p}^{s}\right)$	0
